# One hundred years of change?

**DOI:** 10.1113/EP092536

**Published:** 2025-05-05

**Authors:** Martha C. Tissot van Patot

**Affiliations:** ^1^ Fort Collins Colorado USA

1

I have spent the last 14 years researching and writing about Mabel Purefoy FitzGerald (1872–1973) and her remarkable life in science at the turn of the 20th century. Her adventures led her from the hallowed halls of Oxford to bustling laboratories in New York City, remote and rugged mining camps high in the Rocky Mountains, and on social outings with the Roosevelts. I must have looked like a madwoman, seated for days on end in the Bodleian Library in Oxford, engrossed in the archive of her personal and professional papers, my jaw working, fists clenching and unclenching, and possibly even emitting small growls of sisterly scientific commiseration as I uncovered the shocking trials and tribulations she faced. (Despite academic convention, I prefer to use her first name to foster greater empathy for her experiences.)

Between 1896 and 1933, Mabel accumulated over 3500 h of medical education at six institutions across four countries. She published eleven research articles, including groundbreaking work on the control of respiration and the origin of acid in the stomach. She also trained in clinical medicine with Sir William Osler, the ‘Father of Modern Medicine’, and served as a clinical pathologist for 5 years. She did not receive a university degree until 1972 when the University of Oxford awarded her an Honorary Master of Arts. She was 100 years old.

I was astonished to discover that, although Mabel was a prolific correspondent, there is not a single letter in which she complained that the challenges she faced were because she was a woman. In fact, amidst the pomp and circumstance of receiving her honorary degree, Mabel told a reporter that she never received a degree during her career because she had to stop taking classes to return to Oxford to care for her ageing sisters. At the time, she was 63 years old and still attending classes to fulfil the demands of the London College of Physicians and Surgeons.

Her calm acceptance of her fate prompted me to reflect on my own career in science 100 years later. Like Mabel, throughout my career, I refused to consider that some of my challenges might have been due to sex discrimination. Thus, I began to compare my experiences with Mabel's.

Although societal expectations for women differed for Mabel and me, they significantly influenced our pursuit of scientific careers. In the late 1800s, women were explicitly expected *not* to show any interest in science or medicine. As a result, Mabel's pursuit of a medical degree was fraught with difficulties. In the 1970s and early 1980s, as a young woman in secondary school with an aptitude for science, I was encouraged to become a doctor. However, no one mentioned many other careers in science, including engineering, mathematics, physics, biology, ecology, chemistry or medical research.

Like Mabel, I had excellent early mentors. Sadly, neither of us had female mentors. Our respective mentors helped us thwart the societal expectations placed on us 100 years apart. When Mabel could not get a degree and become a physician because women were not awarded degrees at the University of Oxford, her mentors offered her research positions in their laboratories so that she could follow her dream of studying science. In my case, when one particularly astute undergraduate professor invited me to think about my daily life as a doctor, an image of windowless rooms occupied by sick people sprang to mind, abruptly ending my pursuit of a medical degree. This noble path was not for me. I eventually found my way to a career in biomedical research.

With such supportive mentors early in our careers, what made Mabel's pursuit of a medical degree so fraught with difficulties and my research career so untenable that I had to leave the field early?

We each had a few individuals in our lives who were, at best, less than supportive. In Mabel's case, her mentors at the Rockefeller Institute, director Simon Flexner and researcher Hideyo Noguchi, made her life a misery. They assigned her a project well below her expertise, offered little mentorship, and did not allow women to socialize with their male colleagues, putting them at a great disadvantage in forwarding their research endeavours. In my case, a few department chairs and faculty members significantly increased the challenges of being an academic research scientist. For instance, my equipment was taken away and given to others, my technicians were made to work for other investigators, my name was removed from a grant I wrote that was subsequently funded, and I was compelled to include someone as an author on a publication they had no role in producing.

However, it was ultimately the faceless, nameless boards and committees, whose members bore no personal responsibility for their decisions, that failed us both. In Mabel's case, the admissions committee at Cornell University Medical School, the New York Board of Regents, and the Board of the London Royal College of Physicians created an impossible set of standards she could not overcome. In my case, funding agencies and review committees contributed to my eventual departure. The National Institutes of Health was funding female investigators at a much lower rate than male investigators, and women's health issues (my primary research focused on placentation) received only about 8% of all research dollars. I trusted the scientific integrity of funding review committees throughout my career, refusing to believe that sex, race, gender or personal disputes would influence funding decisions. However, toward the end of my academic career, my confidence in the committees was sorely tested when my proposal to investigate the role of placental mitochondria in hypoxic and ischaemic pregnancy complications was rejected with the rationale that ‘we already know what mitochondria do’. Bias is more easily acted upon when people are part of committees and are not held directly accountable for their actions.

When Mabel's dream job as a clinical pathologist ended because the man she had replaced returned from the war, her mentors and colleagues encouraged her to apply for a position as a lecturer in bacteriology and helped ensure she was awarded the job. Ultimately, she left science because she believed it was her responsibility to care for her ageing sisters, another societal expectation. Unlike Mabel, I did not have mentors to advocate for me, so when funding was difficult and working with aggressive and antagonistic male colleagues became untenable, I left my academic research position.

Although these are only two stories in a vast sea of narratives about women in science, their implications are surprising and concerning. While committees and boards remain as irresponsible as ever, it appears that colleagues and mentors were much more supportive a century ago than they are today. Nevertheless, the world is constantly evolving, and to truly understand the current state of affairs, we need to hear from women who are in the early stages of their scientific careers. Where have you found support and faced challenges? Do you think there are realistic opportunities to foster a more inclusive and supportive scientific community?

## AUTHOR CONTRIBUTIONS

Sole author.

## CONFLICT OF INTEREST

None declared.

## FUNDING INFORMATION

No funding was received for this work.

